# NT5E upregulation in head and neck squamous cell carcinoma: A novel biomarker on cancer-associated fibroblasts for predicting immunosuppressive tumor microenvironment

**DOI:** 10.3389/fimmu.2022.975847

**Published:** 2022-08-26

**Authors:** Xue-min Chen, Yu-yang Liu, Bing-yan Tao, Xin-miao Xue, Xin-xin Zhang, Lin-lin Wang, Hui Zhong, Jun Zhang, Shi-ming Yang, Qing-qing Jiang

**Affiliations:** ^1^ Medical School of Chinese People’s Liberation Army (PLA), Beijing, China; ^2^ Senior Department of Otolaryngology-Head & Neck Surgery, Chinese People’s Liberation Army (PLA) General Hospital, National Clinical Research Center for Otolaryngologic Diseases, State Key Lab of Hearing Science, Beijing Key Lab of Hearing Impairment Prevention and Treatment, Ministry of Education, Beijing, China; ^3^ Department of Neurosurgery, Chinese People’s Liberation Army (PLA) General Hospital, Beijing, China; ^4^ Southern Medical University, Guangzhou, China

**Keywords:** NT5E, head and neck squamous cell carcinoma, tumor microenvironment, cancer-associated fibroblast, extracellular matrix, immunotherapy

## Abstract

Despite tremendous progress made in the diagnosis and managements, head and neck squamous cell carcinoma (HNSC) remains a global medical dilemma with dismal clinical prognosis and high mortality. Gene NT5E encodes the ecto-5’-nucleotidase (CD73), which facilitates the formation of immunosuppressive tumor microenvironment (TME) permissive for tumor progression in various malignancies. Nevertheless, the cell subsets NT5E expressed on and the potential function of NT5E in the TME of HNSC remain virgin lands in HNSC. In this study, we comprehensively performed integrated prognostic analysis and elucidated that NT5E was an independent prognostic indicator for HNSC, for which a high NT5E level predicted poor overall survival (OS), disease-specific survival (DSS) and progression-free interval (PFI) in HNSC patients (*p*<0.05). Enrichment analyses revealed the close correlation between NT5E and ECM remodeling, and the latent function of NT5E may involve in epithelial-to-mesenchymal transition (EMT) and metastasis during HNSC progression. HNSC-related immune infiltration analysis and single-cell type analysis demonstrated that NT5E expression was significantly positively associated with cancer-associated fibroblasts (CAFs) in HNSC (*p*<0.01). NT5E-related TME analysis revealed that NT5E-high group are characterized by low neoantigen loads (NAL, *p*<0.001) and tumor mutation burden (TMB, *p*<0.01), indicating high-NT5E-expression HNSC patients may be recalcitrant to immunotherapy. *In-situ* multicolor immunofluorescence staining was later conducted and the results further verified our findings. Taken together, NT5E could be a novel biomarker in HNSC. Predominantly expressed on CAFs, the upregulation of NT5E might predict an immunosuppressive TME for HNSC patients who may benefit little from immunotherapy. Targeting CAFs with high NT5E expression might be a novel therapeutic strategy for HNSC patients.

## Introduction

According to Global Cancer Statistics 2020 (1), head and neck cancers are amongst the six most common cancers worldwide. In China in 2022, it is predicted that 139,170 new cases of head and neck cancer will be diagnosed, with approximately 75,640 deaths related to these diseases (2). Head and neck squamous cell carcinoma (HNSC), which arises from the epithelial linings of the oral cavity, oropharynx, paranasal sinuses, nasopharynx, larynx, and hypopharynx, accounts for over 90% of head and neck cancers (3–6). There are several potential risk factors that are related to the pathogenesis of HNSC, including cigarette smoking, alcohol consumption, betel quid chewing (7), human papillomavirus (HPV) infection (8) and poor oral hygiene (9). Early HNSC is mainly treated by surgery or radiotherapy, and for intermediate and locally advanced HNSC, multidisciplinary treatment including concurrent radiotherapy, immunotargeted therapy and immunotherapy is required (10–12). Nevertheless, metastasis, recurrence and drug resistance are still the main causes of poor 5-year survival rate, which has remained below 60% (13). Therefore, the exploration of effective diagnostic and prognostic biomarkers is pivotal to improve the therapeutic efficacy, long-term survival rate and quality of life of HNSC patients, and plays an important role on promoting the development of HNSC treatment.

In recent years, the concept of tumor microenvironment (TME) has greatly enriched our understanding of tumor. The stromal components of TME consist of a variety of cell types, such as cancer-associated fibroblasts (CAFs), macrophages, regulatory T cells (Tregs), myeloid-derived suppressor cells (MDSCs), natural killer cells (NK), and mast cells (14). These subpopulations secrete cytokines, chemokine, growth factors and extracellular matrix (ECM) proteins, constructing a cross-linked signaling network by interacting with each other as well as cancer cells. Therein, CAFs with tumor-promoting functions are the major producers of ECM-degrading proteases (matrix metalloproteinases, MMPs), ECM components and various other secreted factors, facilitating the formation of immunosuppressive TME that is permissive for tumor progression (15, 16). During the progress of ECM remodeling, the deposit of collagens IV, VII, XI, and XV is promoted (17), and thus the ECM contractility and stromal stiffness are augmented by CAFs, which is correlated to promoted tumor malignancy and poor patient prognosis (18). Besides, CAFs can secrete transforming growth factor-β (TGF-β), epidermal growth factor receptor (EGFR), hepatocyte growth factor (HGF), insulin-like growth factor (IGF), vascular endothelial growth factor (VEGF), C–C chemokine ligand 2 (CCL2), C–C chemokine ligand 5 (CCL5), C-X-C chemokine ligand 12 (CXCL12), and other active factors (19), and then act on tumor cells *via* paracrine or juxtacrine to activate a variety of important intracellular signal transduction pathways including TGF-β/Smad (20), PI3K/Akt/mTOR (21), Wnt/β-catenin (22), IL-6/STAT3 (23) and Notch (24), thereby triggering epithelial-to-mesenchymal transition (EMT) in tumor cells (25). The expression of E-cadherin, tight junction protein 1 (ZO-1), cytokeratin (CK) and other cell adhesion molecules in epithelial tissues is inhibited, whereas the expression of N-cadherin, vimentin (VIM), α-smooth muscle actin (α-SMA/ACTA2), fibroblast specific protein 1 (FSP1/S100A4) and Osteopontin in mesenchymal cells is induced (26), promoting the loss of epithelial apical-basal polarity, and driving the acquisition of a mesenchymal and motile phenotype (27). The detachment from normal ECM or adhesion to atypical or unfamiliar ECM can lead to a special type of apoptotic cell death called anoikis (28), and CAF-mediated inhibition of anoikis is of great significance to the metastasis of tumor cells (29).

Gene NT5E encodes the ecto-5’-nucleotidase (CD73), which is a glycosyl-phosphatidylinositol (GPI) anchored cell surface enzyme that catalyzes the dephosphorylation of nucleoside 5’-monophosphates, such as adenosine 5’-monophosphate (AMP), converting it into adenosine (30–32). Overexpressed adenosine upregulates NT5E and promotes cancer cells invasion and adhesion to ECM, contributing to tumor growth, angiogenesis and metastasis (33, 34), and also helping tumor cells to escape from immune surveillance (35, 36). Notably, CAFs are the prominent NT5E^hi^ cells in the TME of breast cancer (37). Elevated adenosine in the TME leads to overexpressed NT5E on CAFs *via* activation of the adenosine A_2B_ receptor, thereby inciting the adenosine-A_2B_-NT5E feedforward circuit, which enforces the NT5E immune checkpoint to inhibit immune activation (38). As reviewed previously, NT5E has close correlation of various immune cells such as lymphocytes, macrophages, NK cells, Tregs, dendritic cells, neutrophils, and endothelial cells, affecting the immune homeostasis (39–41). Studies have found that NT5E-mediated immunosuppression might be a potential target for innovative therapeutics in hepatocellular carcinoma cancer (42), breast cancer (43), cervical cancer (44), non-small cell lung cancer (45), glioblastoma (46), pancreatic cancer (47), and gastrointestinal cancer (48). However, the interacting role of gene NT5E and CAFs within the TME of HNSC patients remains unspecified.

In this study, we explored the NT5E-related TME features and intimate relationship between NT5E and CAFs, so as to provide reference for prognosis and selection of new therapeutic targets for HNSC patients, which is a virgin land in HNSC.

## Materials and methods

### Dataset collection and normalization

The RNA-seq data of tumor and paired-normal tissue were downloaded from the TCGA database (https://portal.gdc.cancer.gov/). For unpaired analysis, RNA-seq data of tumor (from TCGA database) and normal tissue (from GTEx database) were obtained from the UCSC XENA database (https://xenabrowser.net/datapages/) (49). Clinical information of head and neck squamous cell carcinoma (HNSC) were downloaded from the TCGA-HNSC dataset (https://portal.gdc.cancer.gov/projects/TCGA-HNSC), and corresponding prognostic information was obtained from Liu et al. (50). The raw data were normalized with the transcripts per million (TPM) method, and log_2_ (TPM+1) transformation was applied for the subsequent analyses.

### NT5E expression analysis

The expression analysis of NT5E was constructed using the R software (Version 3.6.3) and “ggplot2” package was adopted for visualization. The cell type-level expression analysis was performed *via* the GEPIA2021 database (http://gepia2021.cancer-pku.cn/) (51). Furthermore, the representative immunohistochemistry (IHC) staining of NT5E was retrieved from the Human Protein Atlas (HPA) database (http://www.proteinatlas.org) (52).

### Survival analysis

Kaplan-Meier survival analysis was used to determine the association of NT5E expression level with overall survival (OS), disease-specific survival (DSS) and progression-free interval (PFI) in HNSC patients. The HNSC cohort was divided into two groups by median NT5E expression level (high-expression group: 50%-100%; low-expression group: 0%-50%). To further evaluate the prognostic value of NT5E, we also performed subgroup analyses on OS, DSS and PFI in HNSC patients that were stratified by different clinical characteristics. The log-rank test was utilized to verify the difference. The “survival” package was applied for statistical analysis and the “survminer” package was used for visualization.

### Univariate and multivariate Cox regression analysis

To evaluate whether the high expression level of NT5E was an independent prognostic indicator, Cox proportion hazard regression analyses were constructed on TCGA-HNSC database. Firstly, we performed univariate Cox regression analysis and potentially confounding characteristics were selected with *p*<0.1. Secondly, multivariable Cox regression analysis concerning OS, DSS and PFI was used to confirm whether NT5E was an independent indicator. The “survival” package was utilized for statistical analysis. The aforementioned results were manifested as forest plots using the R software (Version 3.6.3) and “ggplot2” package.

### Construction of co-expression network of NT5E

To better understand biological function of NT5E in HNSC, we used the correlation analysis to construct the co-expression network of NT5E in TCGA-HNSC cohort. Top 50 co-expression genes positively and negatively correlated with NT5E were selected for subsequent analyses. The results were displayed in heatmaps and “ggplot2” package was used for visualization.

### Enrichment analysis of NT5E

The Gene Ontology (GO) and Kyoto Encyclopedia of Genes and Genomes (KEGG) enrichment analyses were performed based on top 50 co-expression genes positively and negatively correlated with NT5E. The “clusterProfiler” package was used for statistical analysis and visualization (53). Gene Set Enrichment Analysis (GSEA) based on NT5E was performed on the CAMOIP database (54) (http://www.camoip.net/) and the results of analysis were directly downloaded from it.

### Single cell sequencing data analysis of NT5E

In order to deeply explore the latent function of NT5E on single-cell level, the CancerSEA (55) (http://biocc.hrbmu.edu.cn/CancerSEA/home.jsp) database was used to perform correlation analysis between NT5E expression level and different tumor functional status. The HPA database was used to identify potential cell cluster that expressed NT5E (52). Furthermore, GO treemap was chosen to show the enrichment of GO-terms in the cluster. All the results were automatically generated from online database.

### NT5E-related tumor microenvironment analysis

The immune infiltration was estimated by TIMER2.0 database (56) (http://timer.comp-genomics.org/). For NT5E-related immune infiltration analysis in HNSC, we used EPIC (57) and MCP-counter (58) methods. To estimate the relationship between NT5E and CAFs, a pan-cancer analysis was performed using EPIC, MCP-counter and TIDE (59) methods. All these results were directly generated from TIMER2.0 database. According to the instruction of TIMER2.0, MCP-counter and TIDE were recommended to select the “Purity Adjustment” option. Additionally, there is no need to adjust purity for the association analysis using the estimations from EPIC. To further disclose the association between NT5E and CAF, correlation analyses were performed between NT5E expression level and CAF-related markers collected from Nurmik et al. (60). Additionally, the expression level of CAF-related markers in different groups (NT5E-high and NT5E-low group) was also investigated.

The CAMOIP database was used to further estimate TME of HNSC (54). Immunogenicity-related indicators [neoantigen loads (NAL), tumor mutation burden (TMB), and MANTIS Score] were chosen to predict the potential response to immune checkpoint therapy (61). The immune scores, including stromal fraction, lymphocyte infiltration signature score, TGF-β response, proliferation and macrophage regulation were also analyzed between the NT5E-high and NT5E-low group of HNSC patients.

### Sample collection and immunofluorescence staining

Clinical HNSC samples were collected from inpatients in Senior Department of Otolaryngology-Head & Neck Surgery from Chinese PLA General Hospital, which was approved by the Ethics Committee of Chinese PLA General Hospital (No. S2021-339-02). All of the patients or their legal guardians gave their informed consent to participate. The samples were formalin-fixed and paraffin-embedded, and then were sliced into 3 mm sections. Slides were treated with citrate-EDTA antigen retrieval solution (P0086, Beyotime, China) in a water bath at 100°C for 15 minutes. Subsequently, 10% donkey serum (D9663, Sigma-Aldrich, USA) containing 0.2% Triton X-100 (X100, Sigma-Aldrich, USA) was added for blocking at room temperature. The primary antibody of fibroblast activation protein α (FAP) rabbit polyclonal antibody (1:50, AF6858, Beyotime, China) was firstly used to incubate with slices at 4°C overnight, followed by rinsing in PBS three times for 5 minutes each. NT5E mouse monoclonal antibody (1:50, CL488-67789, Proteintech, USA) were then added following the aforementioned procedures. The cy3-labeled goat anti-rabbit IgG (A0516, Beyotime, China) was added and the slides were incubated away from light in a 37°C incubator, followed by rinsing in PBS three times for 5 minutes each and then staining with DAPI (ZLI-9557, Zhongshan Golden Bridge, China). The results were examined manually under fluorescence microscopy (Ti2-U, Nikon, Japan). Whole slide imaging was conducted by Pannoramic scan system (Pannoramic 250 FLASH, 3DHISTECH, Hungary).

### Statistical analysis

The Wilcoxon rank-sum was utilized to detect the statistical significance between NT5E-high and NT5E-low group. Correlations were calculated and evaluated by Spearman’s correlation coefficient. All statistical analyses were performed using R software (version 3.6.3), and a two-tailed *p <*0.05 was considered as the threshold of significance.

## Results

### Expression of NT5E is upregulated in HNSC

**Figure 1:**
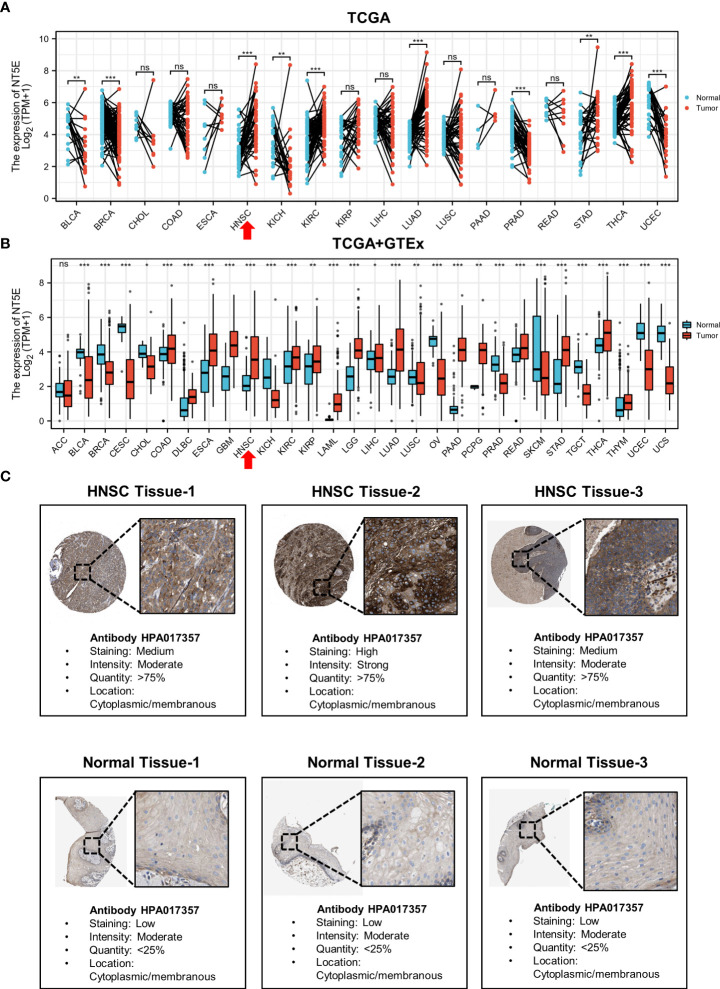
The upregulated expression of NT5E in HNSC. **(A)** The expression distribution of NT5E in tumor and paired-normal tissues (TCGA database). **(B)** The expression distribution of NT5E in tumor and normal tissues (TCGA and GTEx database). **(C)** The expression of NT5E protein in HNSC and normal tissues (HPA database). (ns, *p* ≥ 0.05, *p < 0.05, **p < 0.01, ***p < 0.001).

To illustrate the expression landscape of NT5E, we performed paired and unpaired pan-cancer analysis, the results showed that NT5E expression had significant difference between tumor and normal tissue in a variety of tumors (**Figures 1A, B**). Furthermore, NT5E expression was upregulated in neoplastic sites compared to that of the normal tissues in HNSC (*p* < 0.001) (**Figures 1A, B**). Moreover, the representative picture was picked out from HPA database to manifest *in-situ* NT5E expression, and a prominent higher NT5E expression is displayed in HNSC tissues than that of normal tissues (**Figure 1C**).

### NT5E is an independent prognostic indicator for HNSC

**Figure 2:**
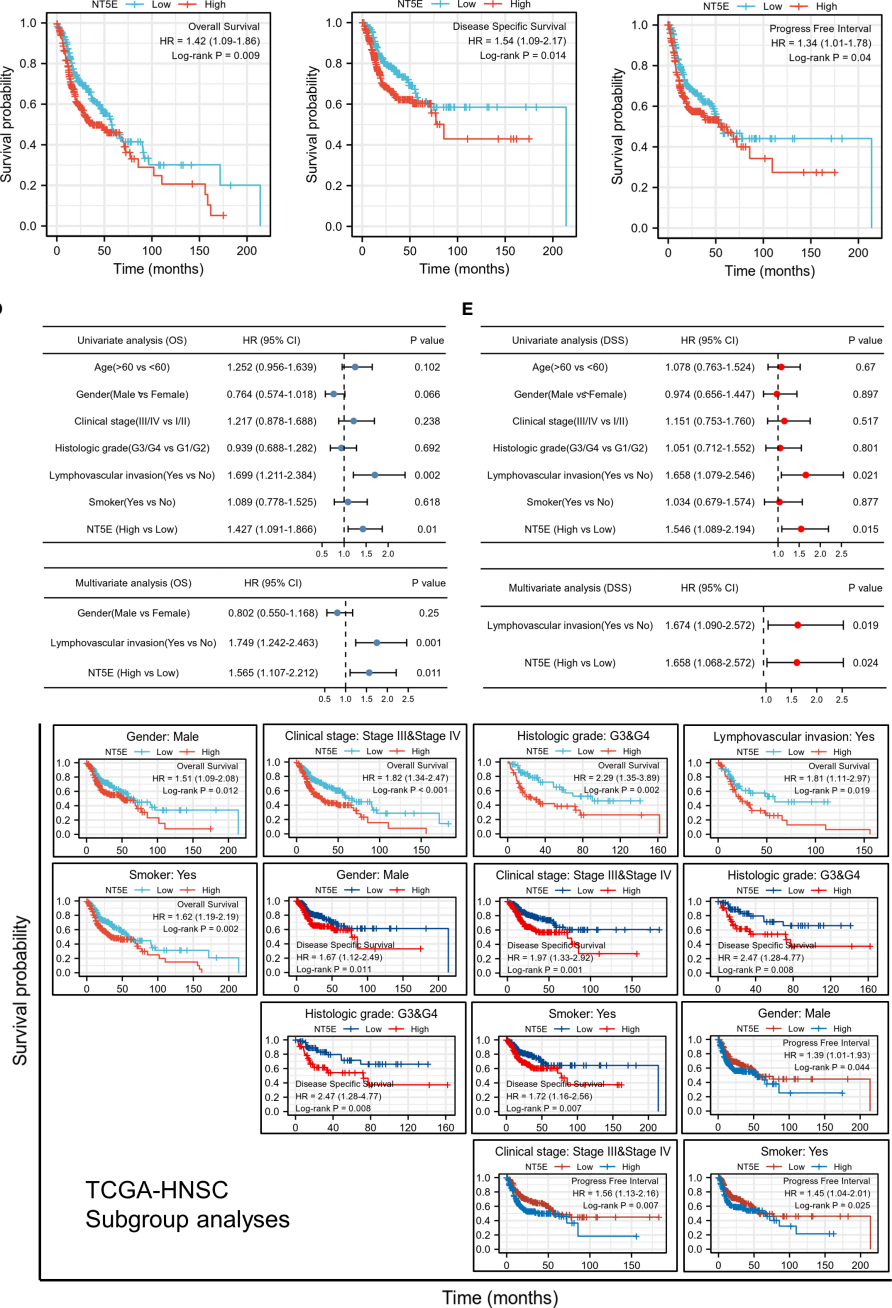
Upregulated expression of NT5E indicates poor prognosis in HNSC patients. **(A)** Patients with high NT5E expression tended to have relatively lower OS (HR=1.42; 95% CI=1.09-1.86; *p*=0.009). **(B)** Patients with high NT5E expression tended to have relatively lower DSS (HR=1.54; 95%CI=1.09-2.17; *p*=0.014). **(C)** Patients with high NT5E expression tended to have relatively lower PFI (HR=1.34; 95%CI=1.01-1.78; *p*=0.04). **(D)** Univariate and multivariate Cox regression analysis of OS (univariate analysis HR=1.427; 95%CI=1.091-1.866; *p*=0.01 and multivariate analysis HR=1.565; 95%CI=1.107-2.212; *p*=0.011). **(E)** Univariate and multivariate Cox regression analysis of DSS (univariate analysis HR=1.546; 95%CI=1.089-2.194; *p*=0.015 and multivariate analysis HR=1.658; 95%CI=1.068-2.572; *p*=0.024). **(F)** Survival analysis of NT5E in different HNSC subgroups.

We examined the predictive value of NT5E in determining clinical prognosis for HNSC patients derived from TCGA database. According to Kaplan-Meier plots, HNSC patients with higher NT5E expression had relatively lower OS [hazard ratio (HR)=1.42; 95% confidence interval (CI)=1.09-1.86; *p*=0.009], DSS (HR=1.54; 95%CI=1.09-2.17; *p*=0.014) and PFI (HR=1.34; 95%CI=1.01-1.78; *p*=0.04) (**Figures 2A–C**). The forest plot of univariate Cox regression analysis indicated that the elevated expression of NT5E could potentially predict unfavorable OS (HR=1.427; 95%CI=1.091-1.866; *p*=0.01), DSS (HR=1.546; 95%CI=1.089-2.194; *p*=0.015) and PFI (HR=1.344; 95%CI=1.012-1.784; *p*=0.041). Confounding characteristics selected with *p*<0.1, we subsequently conducted multivariate Cox regression analysis, and revealed that NT5E may be an independent risk factor for poor prognosis including OS (HR=1.565; 95%CI=1.107-2.212; *p*=0.011), DSS (HR=1.658; 95%CI=1.068-2.572; *p*=0.024) and PFI (HR=1.344; 95%CI=1.012-1.784; *p*=0.041) in HNSC patients. (**Figures 2D, E** and **Supplementary Figure 1**).

### Predictive value of NT5E based on clinicopathologic characteristics

Stratifying HNSC patients by various clinicopathologic characteristics, we investigated the correlations between NT5E expression and survival probability of HNSC patients. The results consistently showed that within the subgroup of gender (male), clinical stage (stage III & IV), histologic grade (G3 & G4), lymphovascular invasion (yes), and smoker (yes), HNSC patients with a higher NT5E expression had a remarkably deteriorative OS and DSS (*p*<0.05). Likewise, within the subgroup of gender (male), lymphovascular invasion (yes), and smoker (yes), high NT5E expression is correlated with unfavorable PFI (*p*<0.05) (**Figure 2F**).

### Enrichment analysis of NT5E manifest its close correlation with ECM

**Figure 3:**
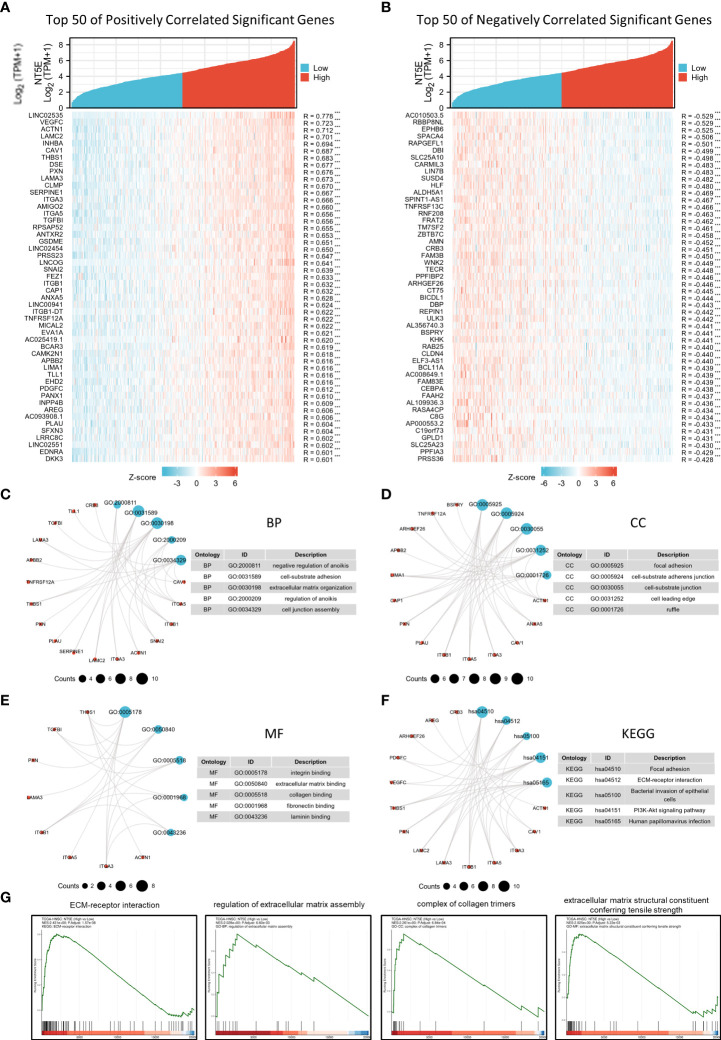
Potential biological function of NT5E in HNSC. **(A)** The top 50 genes positively correlate with NT5E in HNSC (Spearman’s correlation analysis, ***p < 0.001). **(B)** The top 50 genes negatively correlate with NT5E in HNSC (Spearman’s correlation analysis, ***p < 0.001). **(C)** Top 5 BP analyses of NT5E correlated genes in HNSC. **(D)** Top 5 BP CC analyses of NT5E correlated genes in HNSC. **(E)** Top 5 BP MF analyses of NT5E correlated genes in HNSC. **(F)** Top 5 BP KEGG analyses of NT5E correlated genes in HNSC. **(G)** GSEA analyses of NT5E in HNSC.

The correlation analysis was utilized to construct the co-expression network of NT5E in TCGA-HNSC cohort. The heat map showed the top 50 genes positively and negatively correlated with NT5E expression (**Figures 3A, B**). We selected these genes to perform GO and KEGG enrichment analyses. Primary biological process (BP) contained negative regulation of anoikis, cell-substrate adhesion, ECM organization, regulation of anoikis, and cell junction assembly (**Figure 3C**). The cellular component (CC) analyses demonstrated that co-expression genes were enriched in focal adhesion, cell-substrate adherens junction, cell-substrate junction, cell leading edge, and ruffle (**Figure 3D**). The molecular function (MF) was primarily involved in integrin binding, ECM binding, collagen binding, fibronectin binding, and laminin binding (**Figure 3E**). KEGG analysis suggested that focal adhesion, ECM-receptor interaction, bacterial invasion of epithelial cells, PI3K-Akt signaling pathway and HPV infection were potentially associated with NT5E and its correlated genes (**Figure 3F**). In parallel with GO and KEGG analyses, GSEA analysis was also implemented to identify possible biological functions resulting from NT5E upregulation, which indicated that upregulated NT5E expression was associated with enhanced ECM-receptor interaction, regulation of ECM assembly, complex of collagen trimers and ECM structure constituent conferring tensile strength (**Figure 3G**). The abovementioned results highlighted the potential functions of NT5E in ECM remodeling, giving us insights to further explore its biological function in HNSC progressions.

### NT5E may involve in EMT and metastasis during HNSC progression

**Figure 4:**
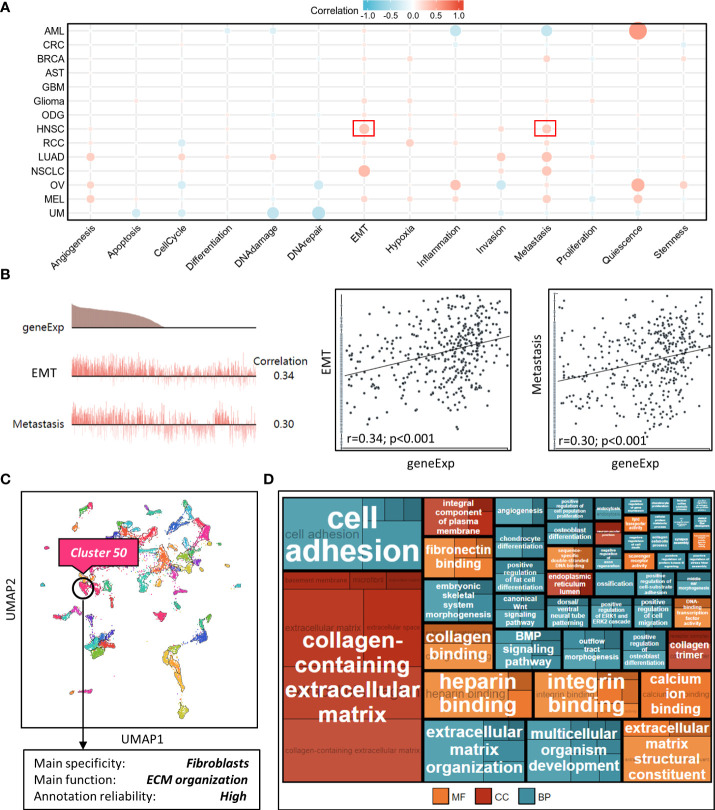
Correlation analysis between NT5E and tumor functional status using single cell sequencing database. **(A)** Heatmap showed the correlation between NT5E expression and different tumor functional status based on the CancerSEA database (Red represents positive correlation and blue represents negative correlation). **(B)** Correlation between NT5E expression and two different functional states in HNSC [r (EMT)=0.34; *p*<0.001 and r(Metastasis)=0.30; *p*<0.001]. **(C)** NT5E expression analysis using UMAP plot from the HPA database and the main specificity of cluster 50 was fibroblasts with ECM organization-related function. **(D)** Enrichment analysis (MF, CC and BP) of cluster 50 based on the HPA database.

We explored NT5E function during HNSC progression on single-cell level from CancerSEA database, and found that among various tumor functional status, it had close relation to EMT and metastasis (**Figures 4A, B**), which might further promote tumor malignancy and exasperating patient prognosis.

### NT5E is closely associated with CAFs in HNSC

**Figure 5:**
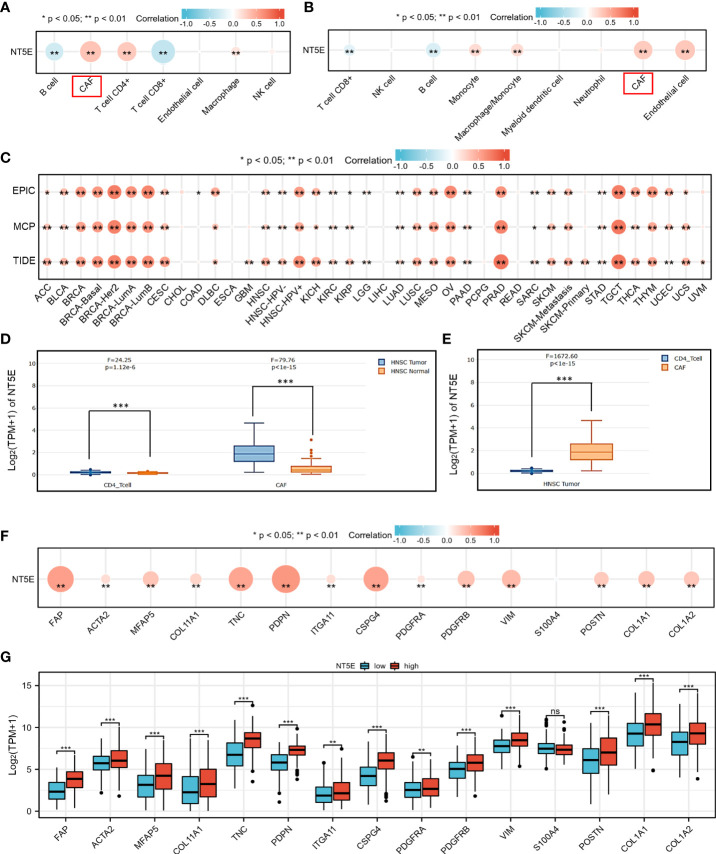
NT5E expression level was associated with unique immune infiltration in HNSC. **(A)** Correlation between NT5E expression and EPIC score of various immune cells. **(B)** Correlation between NT5E expression and MCP-counter score of various immune cells. **(C)** Correlation between NT5E expression and CAF based on TIMER2.0 database. **(D)** NT5E expression patterns of CAF and CD4^+^ T cell based on the GEPIA2021 database. **(E)** Comparison of NT5E expression analysis between CAF and CD4^+^ T cell in HNSC based on the GEPIA2021 database. **(F)** Correlation between NT5E expression and CAF-related markers. **(G)** Comparison of CAF-related markers between NT5E-high and NT5E-low groups. (ns, *p* ≥ 0.05, *p < 0.05, **p < 0.01, ***p < 0.001).

To identify potential cell cluster that expresses NT5E, we utilized the HPA database to construct single-cell type analysis. We found that NT5E expression was enriched in the Cluster 50. According to the automatic analysis of the database, the main specificity of the Cluster 50 was fibroblasts, with high annotation reliability. Subsequent functional analysis indicated the main function of the Cluster 50 was related to ECM organization (**Figure 4C**). Besides, GO analysis was performed afterwards and the results manifested the possible functions of the Cluster 50 (**Figure 4D**), motivating us to do further exploration concerning the association between NT5E and fibroblasts, especially CAFs. HNSC-related immune infiltration analysis *via* EPIC and MCP-counter methods demonstrated that the expression of NT5E was significantly positively correlated with CAFs (R=0.341 and 0.302, respectively; *p*<0.01), CD4^+^ T cells (R=0.309 *via* EPIC methods; *p*<0.01), macrophage (R=0.153 and 0.205, respectively; *p*<0.01), and endothelial cells (R=0.321 *via* MCP-counter methods; *p*<0.01), while negatively associated with B cells (R=-0.293 and -0.24, respectively; *p*<0.01) and CD8^+^ T cells (R=-0.383 and -0.187, respectively; *p*<0.01) (**Figures 5A, B** and **Supplementary Figure 2**). To further substantiate whether NT5E can be deemed as a novel biomarker on CAFs, we conducted pan-cancer analysis and the results were consistent with the former conclusions that NT5E expression was correlated with CAFs in various tumor types including HNSC (**Figure 5C**). The expression of NT5E on CAFs was further verified *via* cell type-level expression analysis and CD4^+^ T cell was chosen as a control because of its positive correlation with NT5E expression according to **Figure 5A**. In neoplastic sites compared to that of the normal tissues in HNSC, NT5E expression is significantly upregulated in CAFs and CD4^+^ T cells (*p*<0.001), whereas the difference degree of NT5E content on CD4^+^ T cells was not as obvious as CAFs (**Figure 5D**). By analyzing specific cell subgroups in HNSC, we also found that the expression of NT5E on CAFs was significantly higher than that of CD4^+^ T cells (**Figure 5E**). In order to do further validation into the association between NT5E and CAFs, correlation analyses were performed between NT5E expression level and CAF-related markers (60). The results showed that NT5E expression was significantly related to FAP (R=0.559; *p*<0.01), ACTA2 (R=0.199; *p*<0.01), microfibrillar-associated protein 5 (MFAP5) (R=0.337; *p*<0.01), tenascin‐C (TNC) (R=0.516; *p*<0.01), podoplanin (PDPN) (R=0.597; *p*<0.01), chondroitin sulfate proteoglycan 4 (CSPG4) (R=0.532; *p*<0.01), platelet derived growth factor receptor beta (PDGFRβ) (R=0.363; *p*<0.01), VIM (R=0.399; *p*<0.01), periostin (POSTN) (R=0.312; *p*<0.01), collagen type I alpha 1 chain (COL1A1) (R=0.348; *p*<0.01), and collagen type I alpha 2 chain (COL1A2) (R=0.329; *p*<0.01) (**Figure 5F**). Besides, NT5E-high group had significantly higher expression of most CAF-related markers compared to that of NT5E-low group (*p*<0.01, **Figure 5G**).

### Upregulated NT5E predicts unique TME in HNSC

**Figure 6:**
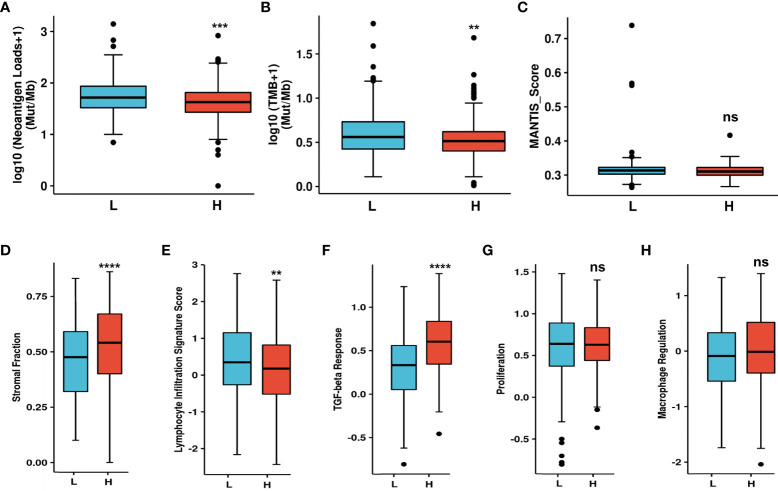
Upregulated NT5E predicts unique tumor microenvironment. **(A)** NAL comparison between NT5E-high and NT5E-low group. **(B)** TMB comparison between NT5E-high and NT5E-low group. **(C)** MANTIS score comparison between NT5E-high and NT5E-low group. **(D)** Stromal fraction comparison between NT5E-high and NT5E-low group. **(E)** Lymphocyte infiltration signature score comparison between NT5E-high and NT5E-low group. **(F)** TGF-beta response comparison between NT5E-high and NT5E-low group. **(G)** Proliferation comparison between NT5E-high and NT5E-low group. **(H)** Macrophage regulation comparison between NT5E-high and NT5E-low group. (ns, *p* ≥ 0.05, **p < 0.01, ***p < 0.001, ****p < 0.0001).

CAFs can secrete various cytokines, inhibit the function of immune cells, regulate ECM remodeling, and thus construct immunosuppressive TME. Accordingly, NT5E-related TME analysis was conducted to further disclose unique TME in HNSC. NAL, TMB and MANTIS Score were chosen to investigate the response to immune checkpoint therapy, and the results showed that the former two had significantly lower rate in NT5E-high group compared to that of NT5E-low group (*p*<0.001 and *p*<0.01, respectively), while the latter one had no significant statistical difference (**Figures 6A–C**). Besides, immune score analysis derived from CAMOIP database was conducted and the results showed that stromal fraction (*p*<0.001), and TGF-β response (*p*<0.001) was higher in NT5E-high group, lymphocyte infiltration signature score (*p*<0.01) was lower in NT5E-high group, comparing to NT5E-low group (**Figures 6D–F**). By contrast, proliferation and macrophage regulation between those two groups had no significant statistical difference (**Figures 6G, H**).

### Validation of NT5E expression pattern on HNSC specimens

**Figure 7:**
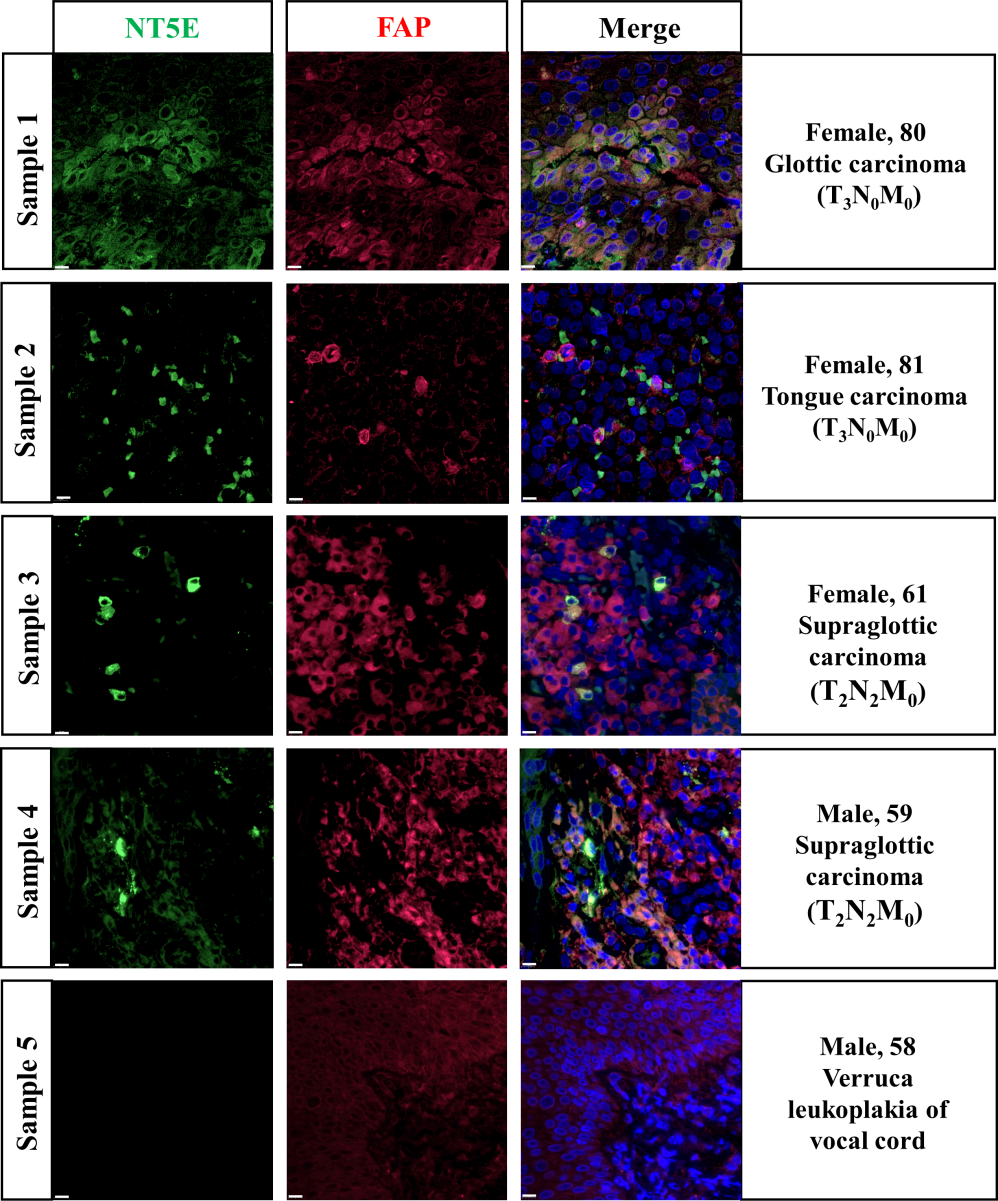
Validation of NT5E expression pattern on HNSC specimens.Scale bar =10 μm. Green stands for NT5E expression, red stands for FAP expression, and blue stands for nuclear staining by DAPI.

*In-situ* immunofluorescence staining was conducted subsequently and the results were shown in **Figure 7**. The pathological diagnoses of Sample 1 and 2 were glottic carcinoma and tongue carcinoma respectively, with TNM-staging of T_3_N_0_M_0_. Sample 3 and 4 were both diagnosed as T_2_N_2_M_0_ supraglottic carcinoma. We found out that in HNSC specimens NT5E was obviously co-localized with FAP, an idealized CAFs marker. Sample 5 was a representative of verruca leukoplakia of vocal cord, who was misdiagnosed as verrucous carcinoma before pathological diagnosis. The staining result demonstrated that the expression of NT5E in non-tumor sample was barely found.

## Discussion

The conventional curative therapies for HNSC are surgical resection, radiation and chemotherapy (9). At present, despite tremendous progress made in the diagnosis and managements, HNSC remains a global medical dilemma with dismal clinical prognosis and high mortality. As no one-size-fits-all treatment strategy is practical for HNSC, screening indicators that adequately reflect the biological characteristics of HNSC and targeting on them would be a novel approach in strategizing the HNSC-patient-specific tailored therapy.

In this study, we comprehensively elucidated the predictive value of NT5E in stratifying clinicopathologic characteristics among HNSC patients *via* bioinformatics methodology. We confirmed that NT5E expression was upregulated in neoplastic sites compared to that of the normal tissues in HNSC. What’s more, by using Cox regression analysis combined with KM survival analysis, NT5E is an independent prognostic indicator for deteriorative OS, DSS, and PFI in HNSC patients. GO and KEGG enrichment analyses combined with GSEA analysis revealed that upregulated NT5E expression was associated with enhanced ECM-receptor interaction, regulation of ECM assembly and ECM binding. The aforementioned findings insinuate that NT5E is involved in ECM remodeling, potentially promoting EMT and metastasis of HNSC tumor cells.

The interrogation into the TME and spatial profiles of HNSC has become a potent tool in understanding cellular interplays which are instrumental in immune checkpoint inhibitors (ICI) and adoptive cellular therapy (ACT) (62). There are three types of the TMEs in various tumors, including immune-desert, immune-excluded and immune-inflamed (63). In the immune-desert TME, immune cells are not able to infiltrate neither the tumor nor the stroma, creating a so-called “cold tumor” environment. Conversely, in the immune-inflamed TME, various types of immune cells infiltrate the tumor and the stroma, and thus is referred to as a “hot tumor”. The immune-excluded TME is a phenotype that occurs when immune cells are restricted to the stroma and are incapable of infiltrating the tumor. On the basis of present knowledge, a subset of HNSC is “immune desert”, which could hijack multiple parts of the tumor immunity cycle so as to suppress immune system activation and evade immune surveillance. According to the analysis of immune infiltration, we uncovered that CD8^+^ T cells in NT5E high group was lower than those of NT5E low group, which was in accordance with the concept of “immune desert”, indicating an immunosuppressive TME.

Previous studies have elucidated that several cell subtypes can express NT5E, including T cells (64, 65), B cells (66), NK cells (67), and CAFs (38, 68) in various tumor types. However, the cell subsets NT5E expressed on and the potential function of NT5E in the TME of HNSC remain elusive. Accordingly, we performed HNSC-related immune infiltration analysis by two distinct methods, and both demonstrated that NT5E expression was significantly positively correlated with CAFs in HNSC. Likewise, single-cell type analysis from HPA database revealed that NT5E expression was enriched in fibroblasts. These results were evidenced by immunofluorescence staining where NT5E and FAP, a CAFs marker, were coexpressed in HNSC samples. However, we still found out some CAFs were FAP-only expressed, so we hypothesized that the NT5E and FAP co-expression pattern may be a unique subpopulation of CAFs. This finding rendered inspiration to us to make further exploration into the function of NT5E-expressed CAFs in HNSC.

As one of the most predominant stromal components in the TME, CAFs may induce immunoinhibitory mechanisms *via* interacting with tumor-infiltrating immune cells as well as other immune components. For one thing, experimental research have revealed the crosstalk between CAFs and immune cells in TME, including orchestrating the optimal tumor stemness-enhancing microenvironment by shaping MDSCs (69), promoting M2 polarization of macrophages (70), and inducing Tregs infiltration at tumor sites (71). For another, CAFs secret various cytokines [e.g., IL-6, macrophage colony-stimulating factor (M-CSF)], growth factors (TGF-β, EGFR, HGF, IGF, VEGF), chemokines (e.g., CCL2, CCL5, CXCL12), exosomes and other effector molecules [e.g., indoleamine 2,3-dioxygenase (IDO), prostaglandin E2 (PGE2)], consequently shaping an immunosuppressive TME that enables tumor cells to evade surveillance of the immune system (19, 72). Besides, CAFs remodel the ECM by secreting multiple matrix proteins (e.g., fibronectin and collagen I) and producing MMPs (e.g., MMP-1, MMP-3), facilitating the degradation of normal ECM structure along with increasing matrix stiffness, so as to boost tumor cell proliferation, angiogenesis, and immune suppression (17, 73). Metabolites such as lactic acid and pyruvate produced by CAFs through glycolysis form an acidic microenvironment inhibiting the activity of immune cells, which can also be utilized as tumor cell nutrients to support tumor metabolism (74, 75). Targeted on CAFs, we hypothesized that NT5E may function as cancer-promoting effectors during HNSC progression.

As for immunotherapy for HNSC, a phase-3 clinical study held by the US Food Drug Administration and the European Medicines Agency have approved pembrolizumab as the first-line treatment of metastatic/recurrent HNSC patients (76). However, only a fraction of HNSC patients currently benefit from approved immunotherapies (77). As is suggested by the Society for Immunotherapy of Cancer (SITC), apart from identifying appropriate subtypes of HNSC patients suitable for immunotherapy, development of other strategies will be imperative for continued progress in treating patients with this heterogeneous disease (78). NAL and TMB are promising biomarkers for predicting the efficacy of ICIs (79, 80). TMB generally refers to the number of non-synonymous mutations per megabase (Mb) of somatic cells in a specific genomic region (80), which is mainly comprised of missense mutations, synonymous mutations, insertions or deletions, and copy number gains and losses (81). On theoretical grounds, the endogenous T cell compartments, especially CD8^+^ T cells, are capable of recognizing peptide epitopes derived from specific mutations in tumor or viral open reading frames, which are displayed on major histocompatibility complexes (MHCs) on the surface of the malignant tumor cells (82). Therein neoantigens are immunogenic peptides entirely absent from the normal human genome. Buttner et al. (83) have confirmed that the higher TMB, the higher tumor NAL, and the more possibility to a patient benefit from ICI therapy. Unfortunately, our results showed that NAL and TMB were significantly lower in NT5E-high group than NT5E-low group, indicating high-NT5E-expression HNSC patients may be recalcitrant to ICI therapy. The underlying reason of high NT5E expression on CAFs leading to lower NAL might be attributable to the immunosuppressive TME it constructs, which inhibits the ability of antigen-presenting cells (APC) to present neoantigens to the host immune system. Besides, HNSC-related immune infiltration analysis demonstrated that NT5E expression was negatively associated with CD8^+^ T cells. Reduced neoantigen specific CD8^+^ T cells also impair the whole anti-tumor immune response in HNSC. Thus, it is urgent to seek new therapy target to amplify clinical benefit and enhance the antitumor effect. Our results innovatively yield insights into potential therapeutic strategies that target CAFs for HNSC patient treatment with high NT5E expression.

Three principal strategies for CAF-directed anticancer therapy are listed as follows (84): depleting CAFs directly by targeting surface markers such as FAP, ACTA2, PDGFRβ, etc.; targeting crucial signals and effectors of CAFs such as growth factor and chemokine pathways; targeting CAFs-derived ECM proteins such as TNC and MMPs. Mao et al. (19) summarized diverse designed drugs that potentially target CAF-associated effector molecules, signaling pathways and matrix proteins, which the cancer model of HNSC was nowhere to be seen. From another aspect, researches have probed in murine tumor models that anti-CD73 antibody therapy and blockade of A_2A_ receptors potently inhibit outgrowth of NT5E expressing tumors (39, 85–88). Moreover, a phase-1 study of MEDI9447, a human monoclonal antibody that is specific for CD73, is currently on the trail (NCT02503774) to treat patients with colorectal carcinoma (89). Currently, no studies have attempted to target CAFs with high NT5E expression to treat solid tumors. Combined with our results, we innovatively propose that targeting CAFs with high NT5E expression may be a good medicine to restrain the immunosuppressive TME, which may be a boon for HNSC patients.

To the best of our knowledge, we are the most integrated analyses of NT5E with HNSC, covering almost all aspects of prognostic analysis, including OS, DSS, PFI, subgroup analysis and single cell analysis. Still and all, there are several limitations of our research. Firstly, HNSC is of strong heterogeneity deriving from different anatomic sites. The acquired HNSC-related database are mostly comprised of oral cavity and tongue, but for other specific subtypes, only a paucity of data could be available from TCGA database. Secondly, the findings of the current investigation demand clinical trial-based validation in a larger HNSC cohort receiving high-NT5E-expression CAFs-targeting immunotherapies. Thirdly, more functional experiments such as flow cytometry and single cell RNA-seq are needed to further elucidate CAFs and NT5E contents in HNSC. Finally, CAFs-targeting therapies have to address the intractable problem of how to improve the antitumor effect and decrease systematic side effects at the same time.

## Data availability statement

The raw data supporting the conclusions of this article will be made available by the authors, without undue reservation.

## Ethics statement

The studies involving human participants were reviewed and approved by the Ethics Committee of Chinese PLA General Hospital. The patients/participants provided their written informed consent to participate in this study.

## Author contributions

X-MC, Y-YL, and B-YT conceived and performed the bioinformatics analysis. Q-QJ, S-MY and JZ were responsible for the data interpretation. X-MC, Y-YL and X-MX co-wrote the paper. X-XZ and L-LW collected clinical samples. X-MX, L-LW and HZ conducted immunofluorescence staining. X-MC and Y-YL undertook the statistical analyses. All authors contributed to and revised the final manuscript.

## Funding

This work was supported by grants from the National Natural Science Foundation of China (No. 82000981 and 81970897) and the National Key Research and Development Program of China (No. 2019YFC0840707).

## Acknowledgments

Thanks are due to Prof. Wei-dong Shen, Prof. Ning Yu, Prof. Wei-wei Guo, Dr. Nan-xiang Chen and Dr. Xiao-chen Ni for their assiatance in clinical sample collection and analysis guidance.

## Conflict of interest

The authors declare that the research was conducted in the absence of any commercial or financial relationships that could be construed as a potential conflict of interest.

## Publisher’s note

All claims expressed in this article are solely those of the authors and do not necessarily represent those of their affiliated organizations, or those of the publisher, the editors and the reviewers. Any product that may be evaluated in this article, or claim that may be made by its manufacturer, is not guaranteed or endorsed by the publisher.

## Supplementary material

Supplementary Figure 1 Univariate and multivariate Cox regression analysis of PFI (univariate analysis HR=1.344; 95%CI=1.012-1.784; *p*=0.041 and multivariate analysis HR=1.344; 95%CI=1.012-1.784; *p*=0.041).

Supplementary Figure 2 Immune infiltration analyses based on NT5E expression level. **(A)** Immune infiltration analysis using the EPIC method. **(B)** Immune infiltration analysis using the MCP-counter method. (ns, *p* ≥ 0.05, **p* < 0.05, ***p* < 0.01, *****p* < 0.0001).
