# Supplementary figures and images for: NT5E upregulation in head and neck squamous cell carcinoma: A novel biomarker on cancer-associated fibroblasts for predicting immunosuppressive tumor microenvironment
Source: Front Immunol. 2022 Aug 26;13:975847. doi: 10.3389/fimmu.2022.975847 (PMC9458906; doi:10.3389/fimmu.2022.975847)

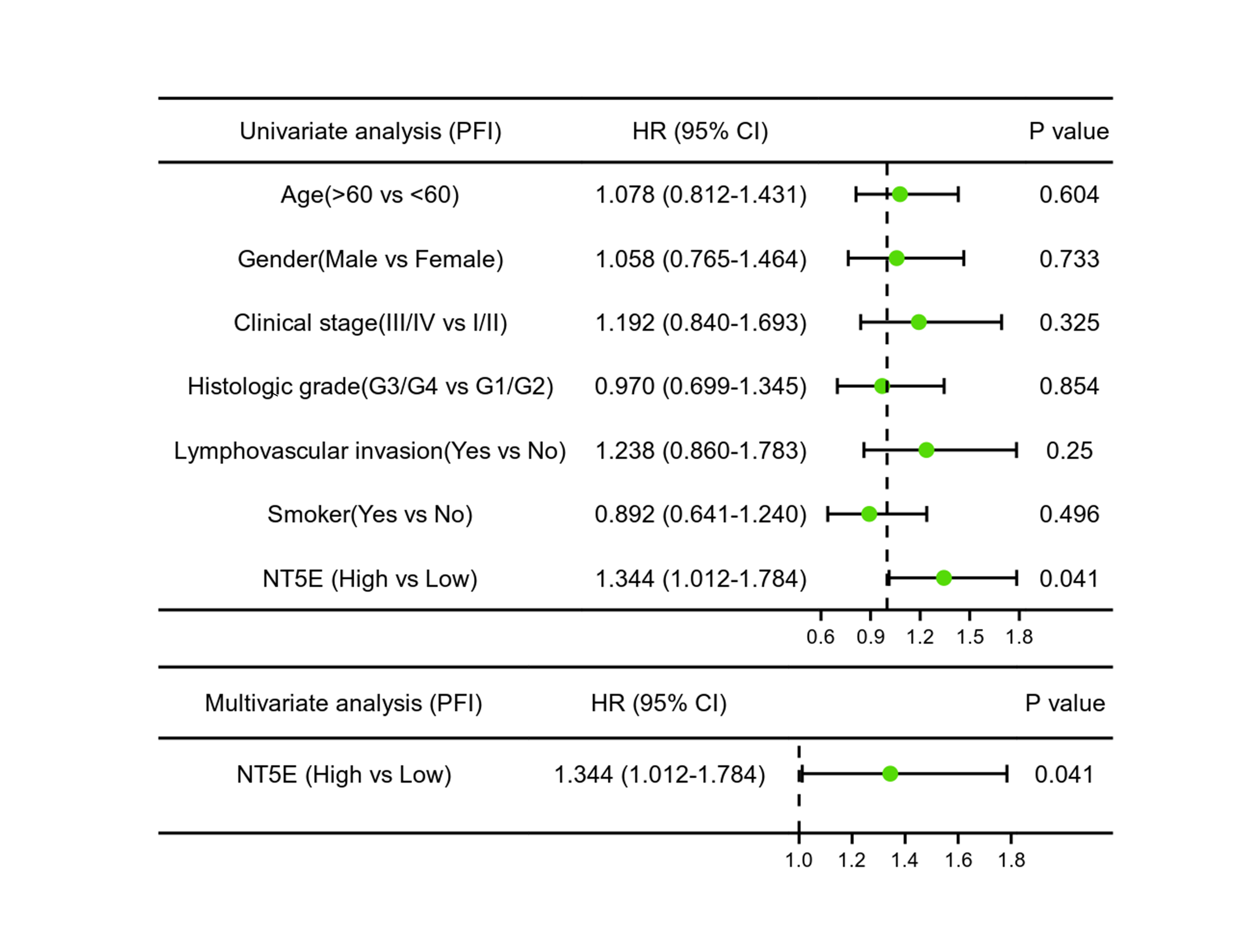

Supplement: Supplementary Figure 1 — Univariate and multivariate Cox regression analysis of PFI (univariate analysis HR=1.344; 95%CI=1.012-1.784; p=0.041 and multivariate analysis HR=1.344; 95%CI=1.012-1.784; p=0.041). [file Image_1.tif]

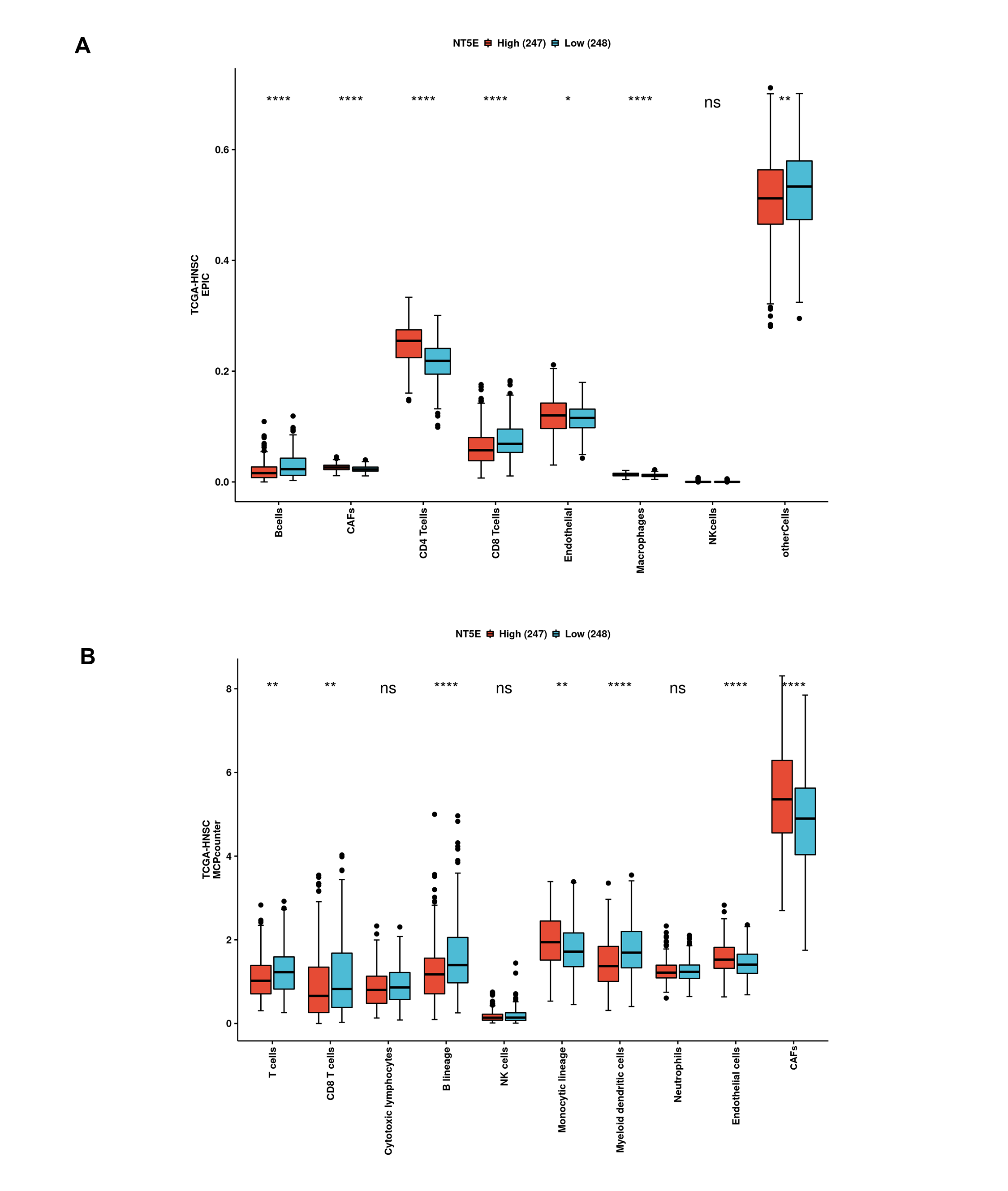

Supplement: Supplementary Figure 2 — Immune infiltration analyses based on NT5E expression level. (A) Immune infiltration analysis using the EPIC method. (B) Immune infiltration analysis using the MCP-counter method. (ns, p ≥ 0.05, *p < 0.05, **p < 0.01, ****p < 0.0001). [file Image_2.tif]
