# Supplementary material for: NT5E upregulation in head and neck squamous cell carcinoma: A novel biomarker on cancer-associated fibroblasts for predicting immunosuppressive tumor microenvironment
Source: Front Immunol. 2022 Aug 26;13:975847. doi: 10.3389/fimmu.2022.975847 (PMC9458906; doi:10.3389/fimmu.2022.975847)
Supplement: Supplementary file 3 [file DataSheet_1.docx]

**Supplementary Materials**

**Table S1. Correlation analysis between CD4^+^ T cells or Tregs cells and NT5E**

| **infiltrates** | **rho** | **p** | **adj.p** |
| --- | --- | --- | --- |
| T cell CD4^+^ (non-regulatory) _QUANTISEQ | 0.13158 | 0.003457 | 0.015695 |
| T cell CD4^+^ (non-regulatory) _XCELL | 0.038259 | 0.397123 | 0.613948 |
| T cell CD4^+^ central memory_XCELL | -0.10969 | 0.014921 | 0.053336 |
| T cell CD4^+^ effector memory_XCELL | -0.01406 | 0.755695 | 0.887966 |
| T cell CD4^+^ memory activated_CIBERSORT | -0.07888 | 0.080492 | 0.186478 |
| T cell CD4^+^ memory activated_CIBERSORT-ABS | -0.07572 | 0.093423 | 0.206158 |
| T cell CD4^+^ memory resting_CIBERSORT | 0.224748 | 4.73E-07 | 6.92E-06 |
| T cell CD4^+^ memory resting_CIBERSORT-ABS | 0.159749 | 0.000375 | 0.002292 |
| T cell CD4^+^ memory_XCELL | 0.102964 | 0.022364 | 0.071821 |
| T cell CD4^+^ naive_CIBERSORT | 0.168522 | 0.000173 | 0.001199 |
| T cell CD4^+^ naive_CIBERSORT-ABS | 0.169531 | 0.000158 | 0.001124 |
| T cell CD4^+^ naive_XCELL | -0.10691 | 0.017682 | 0.060818 |
| T cell CD4^+^ Th1_XCELL | -0.09492 | 0.035306 | 0.100687 |
| T cell CD4^+^ Th2_XCELL | 0.156818 | 0.000481 | 0.002793 |
| *T cell CD4*^+^*_EPIC* | *0.309232* | *4.97E-13* | *1.46E-11* |
| T cell CD4^+^_TIMER | -0.10919 | 0.012552 | 0.040575 |
| **T cell regulatory (Tregs)_CIBERSORT** | **-0.27478** | **5.68E-10** | **1.6E-08** |
| **T cell regulatory (Tregs)_CIBERSORT-ABS** | **-0.24633** | **3.1E-08** | **5.7E-07** |
| **T cell regulatory (Tregs)_QUANTISEQ** | **0.089664** | **0.046836** | **0.125424** |
| **T cell regulatory (Tregs)_XCELL** | **0.052374** | **0.246229** | **0.430356** |

**Table S2. Quantitative analysis for the percentage of CAFs in HNSC specimens**

| **Sample** | **No.** | **FAP^+^**  **number** | **FAP^+^/ Total** | **FAP^+^NT5E^+^**  **number** | **FAP^+^NT5E^+^/ Total** | **Total cell number** | **FAP^+^NT5E^+^/ FAP^+^** |
| --- | --- | --- | --- | --- | --- | --- | --- |
| **Sample1** | **1** | 30 | 25.64% | 5 | 4.27% | 117 | 16.67% |
|  | **2** | 75 | 28.85% | 10 | 3.85% | 260 | 13.33% |
|  | **3** | 72 | 23.84% | 18 | 5.96% | 302 | 25.00% |
|  | **4** | 142 | 39.89% | 25 | 7.02% | 356 | 17.61% |
|  | **5** | 59 | 23.51% | 11 | 4.38% | 251 | 18.64% |
| **Sample2** | **1** | 55 | 20.37% | 12 | 4.44% | 270 | 21.82% |
|  | **2** | 78 | 24.45% | 27 | 8.46% | 319 | 34.62% |
|  | **3** | 24 | 17.14% | 8 | 5.71% | 140 | 33.33% |
|  | **4** | 37 | 16.09% | 10 | 4.35% | 230 | 27.03% |
|  | **5** | 59 | 20.00% | 20 | 6.78% | 295 | 33.90% |
| **Sample3** | **1** | 49 | 22.17% | 10 | 4.52% | 221 | 20.41% |
|  | **2** | 56 | 23.33% | 13 | 5.42% | 240 | 23.21% |
|  | **3** | 20 | 13.70% | 3 | 2.05% | 146 | 15.00% |
|  | **4** | 18 | 9.57% | 5 | 2.66% | 188 | 27.78% |
|  | **5** | 62 | 26.84% | 13 | 5.63% | 231 | 20.97% |
| **Sample4** | **1** | 83 | 25.38% | 24 | 7.34% | 327 | 28.92% |
|  | **2** | 28 | 17.72% | 4 | 2.53% | 158 | 14.29% |
|  | **3** | 35 | 17.95% | 8 | 4.10% | 195 | 22.86% |
|  | **4** | 31 | 14.83% | 12 | 5.74% | 209 | 38.71% |
|  | **5** | 52 | 19.12% | 22 | 8.09% | 272 | 42.31% |
| **Means** | | 53.25 | 21.52% | 13 | 5.17% | 236.35 | 24.82% |
| **Standard Error** | | 28.14 | 0.06 | 7.10 | 0.02 | 64.51 | 0.08 |
